# The Mode of Stem Cell Division Is Dependent on the Differential Interaction of β-Catenin with the Kat3 Coactivators CBP or p300

**DOI:** 10.3390/cancers11070962

**Published:** 2019-07-09

**Authors:** Agnes I. Lukaszewicz, Cu Nguyen, Elizabeth Melendez, David P. Lin, Jia-Ling Teo, Keane K. Y. Lai, Wieland B. Huttner, Song-Hai Shi, Michael Kahn

**Affiliations:** 1 Department of Biochemistry and Molecular Medicine, University of Southern California, Los Angeles, CA 90033, USA; 2 Center for Molecular Pathways and Drug Discovery, University of Southern California, Los Angeles, CA 90033, USA; 3 Department of Molecular Medicine, Beckman Research Institute of City of Hope, Duarte, CA 91010, USA; 4 Department of Pathology, City of Hope National Medical Center, Duarte, CA 91010, USA; 5 City of Hope Comprehensive Cancer Center, Duarte, CA 91010, USA; 6 Max Planck Institute of Molecular Cell Biology and Genetics, Dresden 01307, Germany; 7 Developmental Biology Program, Sloan Kettering Institute, Memorial Sloan Kettering Cancer Center, New York, NY 10065, USA; 8 Department of Molecular Pharmacology and Toxicology, University of Southern California, Los Angeles, CA 90033, USA; 9 Norris Comprehensive Cancer Center, University of Southern California, Los Angeles, CA 90033, USA

**Keywords:** stem cell, asymmetric, symmetric, CBP, p300, p73, β-catenin, ICG-001

## Abstract

Normal long-term repopulating somatic stem cells (SSCs) preferentially divide asymmetrically, with one daughter cell remaining in the niche and the other going on to be a transient amplifying cell required for generating new tissue in homeostatic maintenance and repair processes, whereas cancer stem cells (CSCs) favor symmetric divisions. We have previously proposed that differential β-catenin modulation of transcriptional activity via selective interaction with either the Kat3 coactivator CBP or its closely related paralog p300, regulates symmetric versus asymmetric division in SSCs and CSCs. We have previously demonstrated that SSCs that divide asymmetrically per force retain one of the dividing daughter cells in the stem cell niche, even when treated with specific CBP/β-catenin antagonists, whereas CSCs can be removed from their niche via forced stochastic symmetric differentiative divisions. We now demonstrate that loss of p73 in early corticogenesis biases β-catenin Kat3 coactivator usage and enhances β-catenin/CBP transcription at the expense of β-catenin/p300 transcription. Biased β-catenin coactivator usage has dramatic consequences on the mode of division of neural stem cells (NSCs), but not neurogenic progenitors. The observed increase in symmetric divisions due to enhanced β-catenin/CBP interaction and transcription leads to an immediate increase in NSC symmetric differentiative divisions. Moreover, we demonstrate for the first time that the complex phenotype caused by the loss of p73 can be rescued in utero by treatment with the small-molecule-specific CBP/β-catenin antagonist ICG-001. Taken together, our results demonstrate the causal relationship between the choice of β-catenin Kat3 coactivator and the mode of stem cell division.

## 1. Introduction

Adult somatic stem cells (SSCs) ensure homeostatic maintenance and provide regenerative capacity after insult. Long-term repopulating SSCs, although generally quiescent, can reenter the cell cycle and subsequently, undergo mitosis. These mitotic divisions can be asymmetric, whereby one of the daughter cells remains in the niche as an SSC, while the other daughter proceeds to differentiate. Alternatively, SSCs can undergo symmetric divisions, whereby either both daughter cells remain as SSCs, or both differentiate. The decision to undergo an asymmetric versus a symmetric division is critical both during embryogenesis and in the adult organism. Improper regulation of this decision likely underlies a large array of diseases including cancer, neurodevelopmental and neurodegenerative diseases, and more generally, is associated with the aging process [1,2,3,4].

We have extensively examined the effects and therapeutic potential of selectively antagonizing the CBP/β-catenin interaction, utilizing the specific-small-molecule antagonist ICG-001, in a variety of preclinical models. We have demonstrated the ability to safely eliminate drug-resistant cancer stem/tumor-initiating cells (CSC/TIC), both in solid tumors and leukemias, without any deleterious effects to the normal endogenous stem cell populations [5,6,7,8,9,10,11]. CBP/β-catenin antagonists have also demonstrated efficacy in a variety of injury models including pulmonary and renal fibrosis [12,13], myocardial infarction [14,15] and neurodegeneration [16]. We previously proposed that the beneficial effects of CBP/β-catenin antagonists in these models of regeneration and repair appear are due to enhanced asymmetric differentiation of SSCs and accelerated repair [2]. CSCs intrinsically increase their number of symmetric divisions at the expense of asymmetric divisions due to various mutations (e.g., p53, PTEN, etc.) [2,4,17]. Therefore, the differential effects of CBP/β-catenin antagonists on CSCs versus normal SSCs, forced symmetric differentiation and stochastic elimination versus asymmetric differentiation and enhanced repair without depletion, respectively, must be intrinsically dependent on the targeted cell [1,2,18,19].

**Figure 1:**
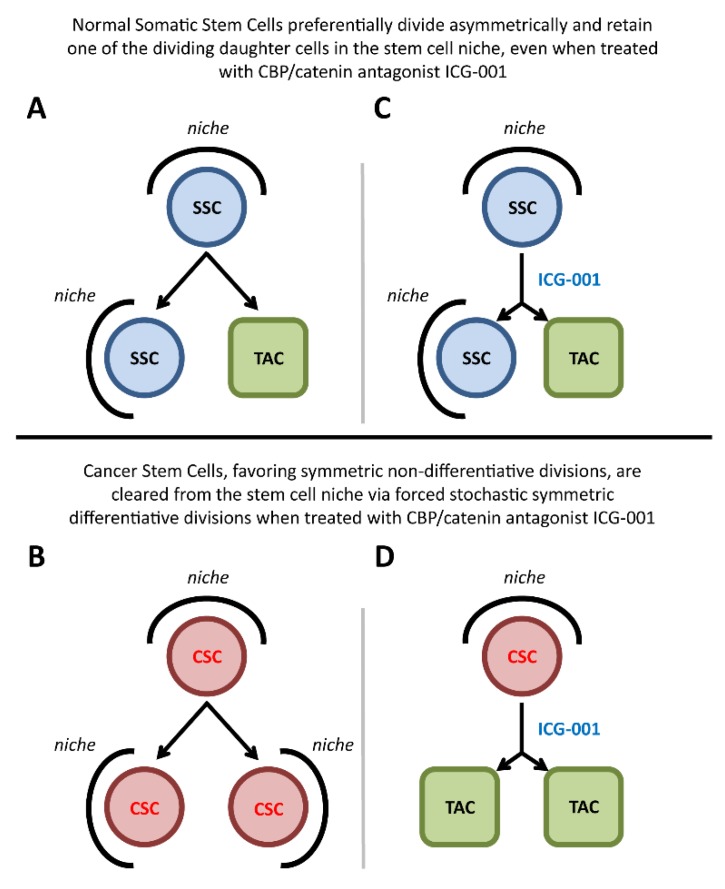
Differential effects of CBP/β-catenin antagonism on cancer stem cells versus normal somatic stem cells. Normal long-term repopulating somatic stem cells (SSCs) preferentially divide asymmetrically, with one daughter cell remaining in the niche and the other going on to be a transient amplifying cell (TAC) required for generating new tissue in homeostatic maintenance and repair processes (**A**), whereas cancer stem cells (CSCs) favor symmetric divisions (**B**). Even when treated with CBP/β-catenin antagonist ICG-001, normal SSCs that divide asymmetrically will always retain one of the dividing daughter cells in the stem cell niche (**C**). However, when treated with ICG-001, CSCs will be removed from their niche via forced stochastic symmetric differentiative divisions (**D**). This fundamental cell-intrinsic difference between CSCs and SSCs thus provides a unique opportunity to therapeutically target and eliminate CSCs, as well as enhance the repair potential of normal SSCs, without damaging or depleting the normal endogenous stem cell populations.

Normal long-term repopulating SSCs preferentially divide asymmetrically, with one daughter cell remaining in the niche and the other going on to be a transient amplifying cell required for generating new tissue both during homeostatic maintenance and repair processes (Figure 1A), whereas CSCs favor symmetric divisions (Figure 1B). However, when treated with CBP/β-catenin antagonists, CSCs will be cleared from their niche via stochastic forced symmetric differentiative divisions (Figure 1D). However, normal SSCs that divide asymmetrically will always retain one of the dividing daughter cells in the stem cell niche (Figure 1C). This fundamental cell-intrinsic difference between CSCs and SSCs thus provides a unique opportunity to therapeutically target and eliminate CSCs, as well as enhance the repair potential of normal SSCs, without damaging or depleting the normal endogenous stem cell pool [2].

To further investigate this model, we chose a system wherein precise regulation of the SSC’s capacity to self-renew and differentiate is necessary for harmonious histogenesis [20]. In this regard, mouse neural stem cells (NSCs) have been extensively and intensively investigated. Symmetric proliferative divisions dominate the earliest stage of neural development. They foster the lateral amplification of the original NSC pool within the germinal zone (GZ) [21]. However, in mice from Embryonic 10.5 days on, NSCs progressively switch to a self-renewing asymmetric mode of division, from which one differentiated daughter cell emerges, i.e., a neuron or an intermediate neuronal progenitor [22,23], and one NSC, thereby critically preserving the NSC pool. Precise regulation of the onset and rate of asymmetric divisions among NSCs allows for the generation of the requisite number of differentiated daughter cells to drive normal CNS development [24].

Subtle changes in the number of progenitors present at the onset of neurogenesis can have a dramatic impact on the expansion of the cortical surface and ultimately, brain size [25,26,27]. Indeed, premature onset of neurogenesis is involved in several neural pathologies including primary microcephalies [28]. Analyses of mouse models for primary microcephaly have established a correlation between neuronal production, NSC maintenance and the mode of division of NSCs [29,30,31,32,33]. Mutations in the microcephalin gene (*MCPH1*) are linked to microcephaly. p73, a member of the p53 family, is a target of MCPH1 [34]. Interestingly, p73, a member of the p53 family, is known to play important roles during CNS development and in the maintenance of CNS homeostasis [35,36,37]. p73 was shown to be required for the maintenance of NSCs during embryogenesis, as its deletion results in the premature depletion of adult neurogenic zones [38,39,40]. Embryonic NSCs isolated from p73 knockout mice (p73KO) show impaired self-renewal capacity and premature differentiation in vitro [38,39,41]. Interestingly, the loss of p73 does not affect NSC specification per se or neuronal commitment [42]. We, therefore, hypothesized that loss of p73 leads to precocious and/or increased neurogenesis at early stages of embryonic development via enhanced β-catenin/CBP transcription and symmetric differentiative divisions with depletion of the NSC pool.

Crosstalk between Wnt- and p73-dependent signaling has previously been examined in both stem cells and more differentiated progenitors [43,44]. We now demonstrate a further mechanistic convergence between the p73 and Wnt/β-catenin pathway in the regulation of the mode of division of embryonic NSCs. We further show that a switch from self-renewing asymmetric to symmetric differentiative divisions in early embryonic NSCs causes the premature depletion of NSCs in p73KO embryos. We biochemically demonstrate that this switch is correlated with differential β-catenin coactivator usage with the Kat3 coactivators, p300 and CBP. Importantly, we demonstrate that this genetic disruption in the mode of division of NSCs can be rescued pharmacologically, with the specific small molecule ICG-001 that enhances the interaction of β-catenin with p300 by specifically antagonizing the CBP/β-catenin interaction. We thereby demonstrate that differential β-catenin Kat3 coactivator usage directly influences the balance between symmetric and asymmetric division in NSCs, and we propose, based on our previous studies, that this extends, more generally, to stem cells.

## 2. Results

### 2.1. Increased Neuronal Production during the Early Stages of Corticogenesis Precedes the Depletion of the NSC Pool in p73KO Mice In Vivo

**Figure 2:**
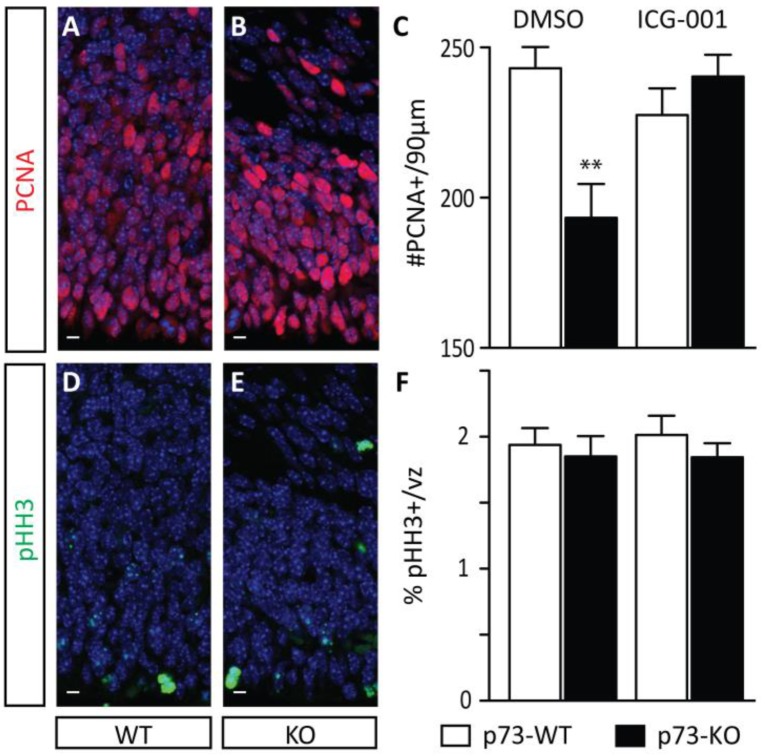
p73 knock-out mouse embryos show depletion of the germinal zone. To monitor the size of the germinal zone, PCNA immunostaining to mark proliferating cells was performed on DMSO-treated p73 wild-type (WT) (**A**) and knock-out (KO) (**B**) embryos (E17.5). A significant decrease in the number of PCNA-positive cells was observed within a 90 µm-wide column in DMSO-treated p73KO embryos compared with their WT littermates, and this decrease was rescued by treatment with CBP/β-catenin antagonist ICG-001 (**C**). To assess the proliferative rate of neurogenic precursors, immunostaining for phospho-histone H3-positive cells (pHH3, a marker of mitotic figures) within the PCNA-positive population was performed in DMSO-treated p73 WT (**D**) and KO (**E**) embryos (E17.5). No difference in the proliferative rate of precursors was observed as quantitated by pHH3 immunostaining (**F**). vz, ventricular zone. *t*-test. *n* ≥ 7. ** *p* < 0.01. scale bar = 10 µm.

The role of p73 during neuronal development has been previously investigated in vitro, implicating p73 in the long-term maintenance of the NSC pool [38,40]. In order to confirm and quantify the phenotype in vivo, we initially monitored the size of the E17.5 germinal zone (GZ). We first performed PCNA (proliferating cell nuclear antigen) immunostaining to mark proliferating cells. As shown in Figure 2, we observed a significant decrease in the number of PCNA-positive cells within a 90 µm-wide column in p73KO embryos compared with their wild-type (WT) littermates (Figure 2A–C: DMSO (dimethyl sulfoxide) control condition). However, the decrease in the overall size of the E17.5 germinal zone did not correlate with a modulation in the proliferative rate of precursors, as assessed by the percentage of phospho-histone H3-positive cells (pHH3+; a marker of mitotic figures) within the PCNA-positive population (Figure 2D–F). This suggested that the observed depletion in the E17.5 germinal zone in p73KO embryos does not per se result from a defect in the proliferative capacities of neurogenic precursors.

**Figure 3:**
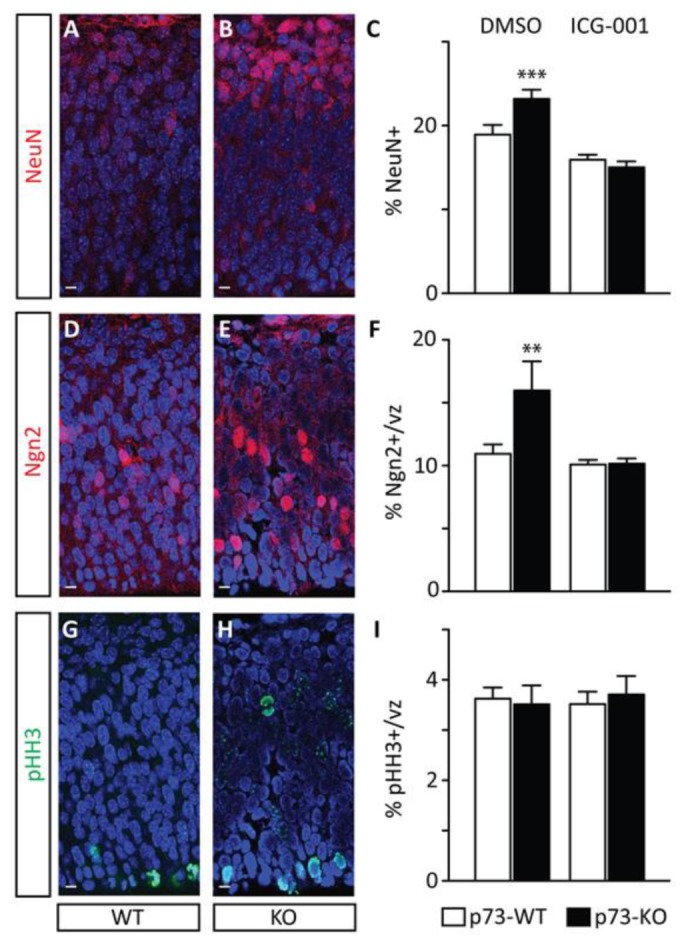
Increased neuronal production during the early stages of corticogenesis precedes the depletion of the NSC pool in p73 knock-out mouse embryos. NeuN immunostaining, a marker of neurogenic status, was performed on DMSO-treated p73 wild-type (WT) (**A**) and knock-out (KO) (**B**) embryos (E13.5). A significant increase in the percentage of post-mitotic neurons as assessed by NeuN positivity was quantitated in DMSO-treated p73 KO embryos compared with their WT littermates, and this increase was rescued by treatment with CBP/β-catenin antagonist ICG-001 (**C**). Adjacent sections of the DMSO-treated p73 WT (**D**) and KO (**E**) embryos (E13.5) were also immunostained for Ngn2, a marker of neurogenic precursors. A significant increase in the percentage of neurogenic precursors as assessed by Ngn2 positivity was observed in DMSO-treated p73 KO embryos compared with their WT littermates, and this increase was rescued by treatment with CBP/β-catenin antagonist ICG-001 (**F**). Using pHH3 as a marker of mitotic cells, no significant difference in mitotic activity was observed between p73 WT and KO embryos (**G**–**I**). vz, ventricular zone. *t*-test. *n* ≥ 6. ** *p* < 0.01, *** *p* < 0.001. scale bar = 10 µm.

To examine if the p73KO phenotype is due to premature neuronal differentiation, we monitored neurogenic status during early corticogenesis using NeuN immunostaining. We first assessed the impact of p73 loss on the post-mitotic neurons present at E13.5. Although the overall cellularity of the cerebral cortex is not affected (Figure S1), E13.5 p73KO embryos present with a significant increase in the percentage of post-mitotic neurons (Figure 3A–C). Adjacent sections were also immunostained for neurogenin 2 (ngn2), a marker of neurogenic precursors. p73KO embryos demonstrate a correlative increase in the percentage of neurogenic precursors present at E13.5 (Figure 3D–F). Taken together, the data reflects an increased rate of neurogenesis in p73KO mice at the early stages of neuronal development. This precocious neurogenesis is manifested in later-stage depletion of the NSC pool.

### 2.2. p73 Loss Induces A Decrease in the Proportion of Self-Renewing Asymmetric Divisions

SSCs self-renew through asymmetric divisions, and can amplify themselves through symmetric proliferative divisions. On the contrary, symmetric differentiative divisions can lead to the exhaustion of the SSC pool [45]. The impairment of NSC maintenance in p73KO embryos in principle could result from either a precocious increase in asymmetric divisions to the detriment of symmetric proliferative divisions during the lateral expansion phase; or enhanced symmetric differentiative divisions at the expense of self-renewing asymmetric divisions during the early neurogenic period [46]. To assess the mode of division of NSCs, we monitored the distribution of the protein Par3, as its pattern of distribution during NSC mitosis has been previously correlated with the mode of NSC division [47]. We focused our analysis on anaphase and telophase mitotic figures, as metaphase spindles are known to undergo dynamic rotation [48]. In WT E13.5 embryos, based on Par3 distribution, we observed that 41.3% of mitoses were asymmetric (Table 1). A striking decrease to 16.7% in the proportion of asymmetric divisions was observed in the p73KO littermates (Table 1). In conjunction with the previously observed concomitant increase in neuronal differentiation, we concluded that loss of p73 leads to the enhancement of symmetric differentiative divisions at the expense of self-renewing asymmetric divisions during the early stages of corticogenesis.

Two additional phenomena could potentially contribute to the depletion of NSCs: (i) a decrease in proliferative potential, pushing progenitors toward premature differentiation [49]; (ii) or increased cell death, within the non-neurogenic pool of precursors, as has been reported at later stages within the p73KO differentiating neuronal population [50,51]. Using pHH3 as a marker of mitotic cells, we were able to demonstrate that there is no significant difference in mitotic activity at E13.5 embryos (Figure 3G–I) or at E17.5 (Figure 2D–F) or increased apoptosis as assessed by caspase-3 associated with the p73KO phenotype. We conclude that the observed increase in early cortical neurogenesis in p73KO embryos reflects aberrations in the mode of division of NSCs, resulting in depletion of the NSC pool.

### 2.3. p73KO Neurogenic Phenotype Is Associated with An Increase in the CBP/β-Catenin Interaction

**Figure 4:**
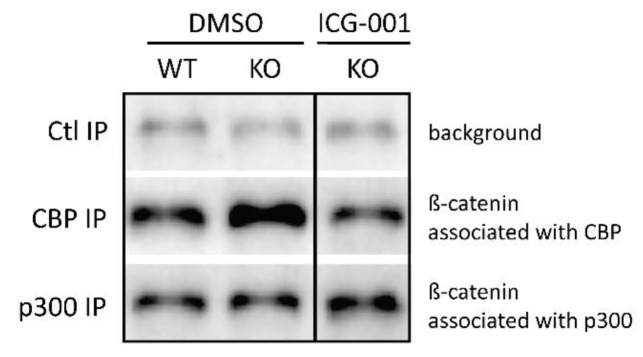
p73 knock-out mouse embryos show an increase in the CBP/β-catenin interaction. Under control conditions, i.e., wild-type DMSO-treated (WT-DMSO) embryos (E13.5), relatively similar levels of β-catenin associated with CBP and p300 were observed. However, knock-out (KO) of p73 led to a dramatic increase in the proportion of β-catenin bound to CBP compared to p300 with DMSO treatment. CBP/β-catenin antagonist ICG-001 treatment normalized the aberrant coactivator distribution interaction nearly back to WT-DMSO levels. IP, immunoprecipitation. Ctl, control antibody. Detailed information can be found at Figure S2.

We previously proposed that differential β-catenin/Kat3 coactivator association plays a key role in the maintenance of SSC pools [1,2,4,19]. Based on our earlier studies, we hypothesized that the enhanced CBP/β-catenin interaction is associated with symmetric divisions and the maintenance of stem cells [52,53] and cancer stem cells [1,2,3,18]; whereas the p300/β-catenin interaction favors asymmetric division and the initiation of differentiation in SSC. To determine if the enhanced symmetric divisions due to p73 loss-of-function correlate with imbalanced β-catenin/coactivator usage, we monitored the relative abundance of β-catenin complexed with CBP or p300. We performed specific immunoprecipitation of either CBP or p300 from E13.5 telencephalon extracts and quantified, via immunoblotting, the presence of bound β-catenin (Figure 4). Under control conditions (WT-DMSO), we observed relatively similar levels of β-catenin associated with CBP and p300. Strikingly, loss of p73 leads to a dramatic increase in the relative levels of β-catenin associated with CBP compared to p300 (2.7 fold). We propose that the disruption of the mode of division in embryonic NSC observed upon p73 loss of function is due to this aberrant increase in the CBP/β-catenin interaction at the expense of p300/β-catenin interaction. To test this hypothesis, we treated mice in utero with the previously well-characterized specific-small-molecule CBP/β-catenin antagonist ICG-001 [54,55]. ICG-001 binds specifically and with high affinity to the N-terminus of CBP, inhibiting its interaction with β-catenin and thereby indirectly increases the interaction between p300 and β-catenin [54]. E9.5 pregnant females were treated daily p.o. (Per Os (oral administration)) with ICG-001 for four days. A co-immunoprecipitation assay demonstrated that ICG-001 treatment pharmacologically reverses the aberrant coactivator distribution interaction in E13.5 telencephalons, resulting in a return to almost WT levels (Figure 4).

Importantly, based upon Par3 distribution, ICG-001 treatment is also sufficient to correct the proportion of asymmetric divisions to the normal level (Table 1, ICG-001-WT: 40.5%, ICG001-KO: 45.8%). Treatment also corrects neurogenesis in the p73KO early cerebral cortex (Figure 2C: ICG-001 treatment); and thereby preserves the NSC pool at later stages of neural development (Figure 3C,F and Figure S1). Taken together, these data demonstrate that aberrantly increased CBP/β-catenin at the expense of the p300/β-catenin interaction is biochemically intrinsically responsible for the disruption of the mode of mitotic division and the subsequent increased premature neurogenic activity of early cortical p73KO NSCs.

### 2.4. p73 Regulates PP4, A Key Factor in Mitotic Spindle Orientation

Our results demonstrate that p73KO disrupts the mode of division of early NSCs. Mitotic spindle orientation appears to play a decisive role in the choice between symmetric and asymmetric division in NSCs during neurogenesis [56,57,58]. Interestingly, p73 transcriptional activity has recently been correlated with the dephosphorylation of critical protein regulators at the mitotic spindle assembly checkpoint [59]. This result suggested that the transcriptional activity of p73 affects the expression or activity of specific phosphatases involved in mitosis. The phosphatase PP4 has recently been linked to the regulation of neurogenesis in the developing cerebral cortex [60,61]. By regulating the phosphorylation status of Ndel1, PP4 modulates Ndel1’s interaction with Lis1, thereby affecting mitotic spindle orientation [60]. PP4c and PP4r3alpha/SMEK1, respectively, the catalytic and a regulatory subunit of the PP4 phosphatase, are both expressed in the developing cerebral cortex as early as E11.5 and localized at centrosomes [60]. The Lis1-Ndel1 complex is essential for radial glial neuroepithelial stem cell self-renewal during the early phases of corticogenesis and defects in this interaction hinder the accurate control of cell division asymmetry and underlie dramatically increased neuronal differentiation [62].

**Figure 5:**
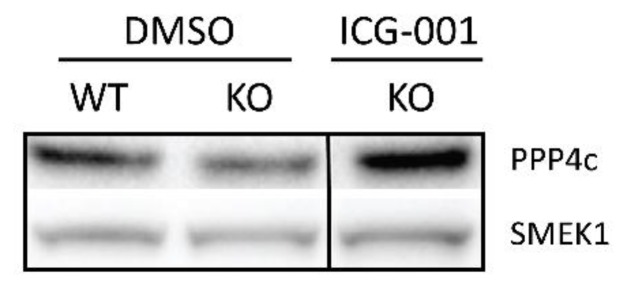
p73 regulates PP4, a key factor in mitotic spindle orientation. Compared to telencephalons from wild-type (WT) mouse embryos (E13.5), those from p73 knock-out (KO) demonstrated lower levels of both PPP4c and its regulatory subunit SMEK1. CBP/β-catenin antagonist ICG-001 treatment rescued the expression of PPP4c and SMEK1. Detailed information can be found at Figure S3.

PP4 has previously been shown to mediate the localization of the Miranda complex during Drosophila neuroblast asymmetric differentiation [63]. Additionally, Wnt signaling has been demonstrated to interact with the Smek/PP4 complex [64]. We thus hypothesized that Wnt/p73 signal transduction may regulate PP4 expression and/or activity. We first compared the expression of PP4c and SMEK1 in the telencephalons of both WT and KO littermates at E13.5 (Figure 5). Interestingly, we observed that the p73KO telencephalons demonstrated lower levels of both PP4c and its regulatory subunit SMEK1 (on average 0.72 and 0.67 of WT, respectively) (Figure 5), which could lead to a reduction in PP4 activity. Since ICG-001 treatment corrected the mode of division in NSCs, we hypothesized that ICG-001 treatment could also rescue the expression of PP4c and SMEK1. Immunoblot analysis confirmed that ICG-001 treatment rescues the levels of both PP4c and SMEK1 essentially back to control levels (both back to 1.04 of WT levels) (Figure 5). To determine whether the expression of PP4c and/or SMEK1 is directly regulated by Wnt/p73, we performed qPCR analysis. Somewhat surprisingly, no difference in the levels of PP4c and SMEK1 mRNA were found comparing WT and KO embryos (Table S1). We conclude that Wnt/p73 signal transduction, perhaps in part through Wnt/STOP signaling, which has been shown to play an important role during cell division [65], regulates mitotic spindle orientation and thereby, the mode of division in NSCs indirectly, at least in part by sustaining the level and activity of the phosphatase PP4.

### 2.5. Specific Blockage of the p300/β-Catenin Interaction Recapitulates the p73KO Phenotype

To further demonstrate the causal relationship between the modulation of β-catenin Kat3 coactivator interactions and the mode of division of embryonic cortical NSCs, we utilized two previously well-characterized specific-small-molecule p300/β-catenin antagonists, YH249 and IQ1 [52,66,67,68]. These molecules, through two independent mechanisms, decrease the interaction between β-catenin and p300, thereby enhancing the CBP/β-catenin interaction and maintaining “stemness” in vitro in a variety of cell types [52,66,67,68] (e.g., mES (murine embryonic stem cells) and SSC, hPSC (human pluripotent stem cells)). YH249, which is structurally related to ICG-001, binds directly and specifically to the N-terminus of p300 thereby, inhibiting its interaction with β-catenin [66]. IQ1, via inhibition of the PR72/130 subunits of the phosphatase PP2A, modulates the phosphorylation status of p300 Ser89 and thereby, its affinity for β-catenin [52,67]. To monitor the consequences of pharmacologic enhancement of the β-catenin/CBP interaction in embryonic NSCs, E9.5 pregnant female wild-type mice were treated daily p.o. with YH249 or IQ1 and E12.5 embryos were collected for analysis. First, we examined the impact of YH249 and IQ1 treatment on the mode of division of NSCs, again using Par3 distribution. As presented in Table 2, both YH249 and IQ1 led to a decrease in the proportion of asymmetric divisions in the E12.5 cerebral cortex (decreased to 6.9% and 22.2%, respectively, compared with 46.4% in the DMSO control). Similar treatment with ICG-001 did not have any significant effect on the mode of division (47.1% asymmetric divisions). However, as previously observed in the p73KO mice, in the wild-type mice, a 3-fold excess ICG-001 significantly rescued the phenotype triggered by YH249 treatment (back to 36.5% of asymmetric divisions) (Table 2).

To further characterize the neurogenic phenotype, we utilized Tis21-GFP mice (GFP knocked-in to the Tis21 ORF (open reading frame) [69], which mark committed neurogenic precursors. Tis21-GFP^+^ precursors have been previously shown to undergo asymmetric divisions [70]. We confirmed that about 2/3 of Tis21-GFP^+^ mitotic figures present in the E12.5 ventricular surface underwent asymmetric mitotic division, based upon Par3 distribution (65.6%; Table 2 last row). Interestingly, YH249 treatment does not significantly affect this committed population (56.7% in YH-249 treated embryos; Table 2). This suggests that differential β-catenin coactivator usage critically and specifically regulates the mode of division in NSCs but less so in more committed progenitors.

**Figure 6:**
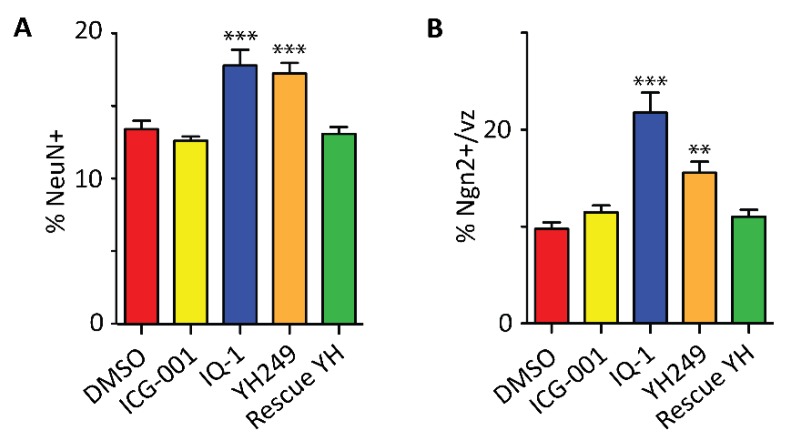
Specific blockage of the p300/β-catenin interaction in wild-type mouse embryos increases the proportions of post-mitotic neurons and neurogenic precursors. To assess the consequences of direct perturbation of the mode of division in NSCs on the rate of neurogenesis, we investigated NeuN and Ngn2 expression by immunostaining. Treatment with small-molecule p300/β-catenin antagonists, YH249 or IQ-1, increased the percentage of post-mitotic neurons (**A**), as well as the percentage of neurogenic precursors (**B**) in wild-type mouse embryos (E12.5), similarly to KO of p73. The effects of YH249 were reversed by ICG-001 treatment (Rescue YH in A-B). One-way ANOVA followed by a Newman–Keuls multiple comparison test showing a significant difference of IQ-1 and YH249 treatments from the other three treatments. vz, ventricular zone. *n* ≥ 6. ** *p* < 0.01, *** *p* < 0.001.

To assess the consequences of direct perturbation of the mode of division in NSCs on the rate of neurogenesis, we again investigated NeuN and ngn2 expression by IF (immunofluorescence). Treatment with YH249 or IQ-1 increased the percentage of post-mitotic neurons, as well as the percentage of neurogenic precursors, similarly to p73KO (Figure 6A,B). Whereas administration of ICG-001 alone did not have any effect on neurogenesis (Figure 6A,B), the prematurely enhanced neurogenic commitment observed with YH249 treatment was reversed by co-administration of ICG-001 (Figure 6A,B). Similar results were obtained using the Tis21-GFP reporter mice to mark neurogenic commitment after IQ1 treatment (Figure S4).

**Figure 7:**
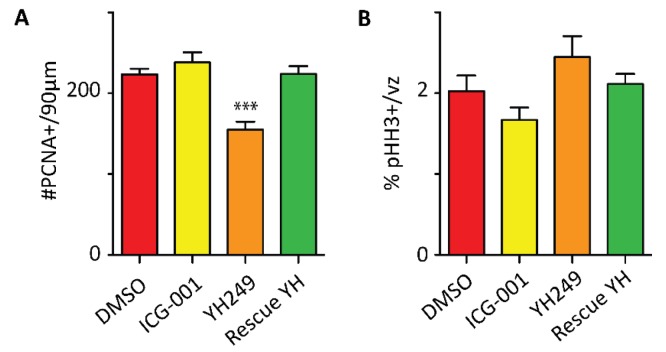
Specific blockage of the p300/β-catenin interaction in wild-type mouse embryos significantly depletes the neural stem cell pool. Treatment with small-molecule p300/β-catenin antagonists, YH249, decreased proliferating cells in embryos (E17.5), as assessed by PCNA immunostaining, and this effect was reversed by ICG-001 treatment (Rescue YH) (**A**). No difference in the proliferative capacity of precursors was observed, as assessed by pHH3 immunostaining (**B**). One-way ANOVA followed by a Newman–Keuls multiple comparison test showing a significant difference (*** *p* < 0.001) of YH249 treatment from the other three treatments. vz, ventricular zone. *n* ≥ 6.

Finally, we monitored the long-term impact of antagonizing the β-catenin/p300 interaction on NSC pool maintenance. E9.5 pregnant wild-type Bl6 females were treated daily with YH249 for 8 days. Recapitulating the effects seen with p73KO embryos, YH249 treatment resulted in significant depletion of the NSC pool in the GZ at E17.5 (Figure 7). Daily treatment with ICG-001 did not have any effect on neurogenesis or the size of the NSC pool, although it was able to rescue the premature depletion of the NSC pool triggered by YH249 (Figure 7).

We conclude that direct disruption of the mode of NSC division via modulation of the β-catenin interaction with p300 affects the rate of neurogenesis at the early stages of cerebral cortex development, which is likely coupled to PP4 regulation of the phosphorylation status of Ndel1 thus modulating its interaction with Lis1, thereby affecting mitotic spindle orientation and the accurate control of cell division asymmetry associated with normal neuronal differentiation [62]. The long-term consequences, as observed in the p73KO mouse include the depletion of the NSC pool at the end of embryogenesis [50]. Taken together, our results demonstrate that either direct pharmacologic inhibition of the β-catenin/p300 interaction or indirect genetic reduction of this interaction via p73 deletion results in premature depletion of the NSC pool via enhanced premature symmetric differentiative divisions. Importantly, both defects can be pharmacologically rescued in utero by utilizing the specific-small-molecule CBP/catenin antagonist ICG-001.

## 3. Discussion

Timely activation and proper control of the mode of division of the SSC pool are critical decisions required during development and for both normal adult homeostasis and repair after injury. We have previously demonstrated that the potent specific-small-molecule CBP/β-catenin antagonist ICG-001, can safely eliminate drug-resistant cancer stem cells (CSCs), yet have beneficial effects on the normal endogenous stem cell populations [5,6,7,8,9,10,11]. To rationalize the beneficial effects of CBP/β-catenin antagonism in these models, we have proposed that the differential effects of CBP/β-catenin antagonists on CSCs versus normal SSCs (i.e., forced differentiation and elimination of CSC versus enhanced differentiation of SSCs and accelerated repair without SSC depletion) must be intrinsically dependent on the targeted cell [1,2,18,19], i.e., taking advantage of the intrinsic propensity of CSCs to increase the number of symmetric divisions at the expense of asymmetric divisions [2,4,17], whereas normal long-term repopulating SSCs preferentially divide asymmetrically. Thereby, CSCs treated with CBP/β-catenin antagonists will be stochastically cleared from their niche via forced symmetric differentiative divisions (Figure 1D), whereas normal SSCs dividing asymmetrically will per force retain one of the dividing daughter cells in the stem cell niche (Figure 1C).

We have now examined this model during mouse embryonic neurogenesis [22]. Previous in vitro and in vivo studies [50] have implicated p73, in the maintenance of the NSC pool during embryogenesis [38,39,41]. We demonstrate that this complex phenotype can be explained by a switch in the mode of division of apical NSCs from asymmetric to symmetric differentiative divisions, which is integrally associated with β-catenin’s choice of transcriptional partners between the two highly homologous Kat3 coactivators, CBP and p300. We biochemically demonstrate that loss of p73 in early corticogenesis biases β-catenin coactivator use and enhances β-catenin/CBP transcription at the expense of β-catenin/p300 transcription (Figure 4). Furthermore, the concomitant increased symmetric differentiative divisions of NSCs in the p73KO is phenocopied by two specific-small-molecule p300/β-catenin antagonists, IQ1 [52] and YH249 [66], which inhibit the p300/β-catenin interaction, thereby enhancing the interaction of β-catenin with CBP. Biased β-catenin coactivator use has dramatic consequences on the mode of division of NSCs, but significantly less so in neurogenic progenitors [71], as quantified by the distribution of Par3 in mitotic NSCs and Tis21-GFP+ neurogenic precursors [47,70,72]. The observed increase in symmetric divisions due to enhanced β-catenin/CBP interaction and transcription leads to an immediate increase in NSC symmetric differentiative divisions and subsequently, long-term depletion of the NSC pool. Furthermore, we demonstrate for the first time that this complex phenotype caused by the KO of p73 can be rescued in utero by treatment with the small-molecule-specific CBP/β-catenin antagonist ICG-001. Taken together, our results demonstrate the causal relationship between the choice of β-catenin Kat3 coactivator use and the mode of division of NSCs and we propose, based on our earlier studies, that this can be extended, more generally, to stem cells [1,2,6].

The data presented here at first glance may appear contradictory to previously published in vitro data. In vitro studies have concluded that the pharmacologic disruption of p300/β-catenin interaction is sufficient to maintain pluripotency and the self-renewal of both mouse and human ES (embryonic stem cell) cells [1,2,52,53,66]. However, in vivo in early embryos, as opposed to in vitro, decreasing the p300/β-catenin interaction leads to enhanced symmetric differentiative divisions in NSCs rather than enhanced symmetric proliferative divisions. Symmetric differentiative divisions naturally occur in the developing mouse cerebral cortex, at the end of the neurogenic period, leading to the depletion of the germinal zone [73]. The decision to symmetrically maintain potency or differentiate is likely context-dependent and not completely cell intrinsic [74]. Indeed, opposite neurogenic outcomes have been previously described from in vivo and in vitro studies and could reflect a change in environmental cues: loss of cell polarity [75], mitogen exposure [76] and feedback mechanisms [77]. Conditionally deleting the Pten tumor suppressor gene in adult hematopoietic cells led to myeloproliferative disease within days and leukemias within weeks and also promoted hematopoietic stem cell (HSC) proliferation. However, this led to premature HSC depletion [45]. Both cell-intrinsic and -extrinsic mechanisms affect the choice between symmetric proliferative divisions in LSC versus symmetric differentiative divisions in the normal HSC population. In this study, we further demonstrated that the modulation of the β-catenin interaction with its Kat3 coactivators has minimal impact on the nearby Tis21-GFP+ neurogenic precursors [71]. Importantly, the specific effects of small-molecule pharmacologic tools on a subset of Wnt/β-catenin transcriptional complexes can assist in dissecting the complexity of this decision process, i.e., cytoskeletal versus transcriptional, and rationalize the seemingly contradictory results often obtained from genetic manipulation of β-catenin [78,79].

Tight control over SSC self-renewal capacity is essential to prevent oncogenesis [45,80,81]. Recently, Lis1, a key factor that regulates several critical aspects during cerebral cortex development and, in particular, the mode of division of NSCs [82], has also been shown to play a role in both normal hematopoiesis, as well as in leukemogenesis [83]. Loss of p53, part of the p53, p63, p73 family of tumor suppressor genes, the most frequently mutated gene in human cancers, favors symmetric divisions of CSCs [84] and has also recently been implicated in the regulation of the mode of division of SSCs during development, via the regulation of β-catenin stability [85]. These pathways, as well as many others, likely play similar and critical roles in the regulation of SSCs both during development and in adult tissue homeostasis. Deciphering the various molecular pathways that regulate the choice between symmetric and asymmetric divisions in SSCs is greatly needed. Comprehension of this critical decision in SSC biology has significant therapeutic implications for genetic disorders, tissue regeneration and repair, aging and cancer.

## 4. Materials and Methods

### 4.1. Mouse Lines and In Utero Treatment by Small Molecules

Animal studies were approved by the USC (University of Southern California) institutional (Institutional Animal Care and Use Committee) IACUC as per protocol #11158 (approval on 24 April 2008). Wild-type (WT), p73 knock-out (KO) [37] and Tis21-GFP [69] mouse lines were used. Pregnant females were treated daily from E9.5 with DMSO vehicle, IQ-1 (half dose and full dose: 72 mg/kg and 144 mg/kg, respectively), YH249 (260 mg/kg) and/or ICG-001 (660 mg/kg) mixed in 2 g of peanut butter. Embryos were collected at E12.5, E13.5 or E17.5. Heads (E12.5 and E13.5) or isolated brains (E17.5) were immersed in PFA (Paraformaldehyde) 4% for 3 h, rinsed in cold PBS (phosphate-buffered saline) and submerged in cold 10% sucrose overnight. Samples were then embedded in OCT (Optimal cutting temperature) compound and kept at –80 °C. A number of 14 µm sections were cut using a Leica cryotome.

### 4.2. Immunofluorescence Staining

After unmasking treatment with DAKO Target Retrieval Solution (#S1700) and blocking with normal serum, the following antibodies were used: Activated Caspase-3 (rabbit polyclonal, R&D Systems, Minneapolis, MN, USA); GFP (chicken polyclonal, Abcam, Cambridge, MA, USA); GFP (rabbit polyclonal, Molecular Probes, Grand Island, NY, USA); mNeuN (mouse monoclonal MAB377, Chemicon, Burlington, MA, USA); mNgn2 (mouse monoclonal, David J. Anderson, California Institute of Technology, Pasadena, CA, USA); Par3 (rabbit polyclonal, Songhai Shi, Memorial Sloan Kettering Cancer Center, New York, NY, USA); PCNA (mouse monoclonal MAB424, Chemicon); phospho-Histone H3 (Ser10; Millipore 06-570); phospho-Vimentin (Ser55; MBL; Woburn, MA, USA; 4A4). DAPI was used as a counter-stain. All pictures were taken using a Zeiss LSM5 confocal system and further analysis of intensity levels was processed using Adobe Photoshop software (San Jose, CA, USA).

### 4.3. Western Blots

Co-immunoprecipitation of CBP or p300 with β-catenin was performed as described elsewhere [52]. For Western blots, the following antibodies were PPP4c (Abcam, ab16475); PPP4R3alpha/SMEK1 (Bethyl, Montgomery, TX, USA; A300-840A). Protein lysates were isolated from E13.5 WT and KO p73 embryos treated for 4 days with DMSO or ICG-001 (660 mg/kg), using the T-Per Tissue Protein Extraction Reagent (Thermo Scientific, Grand Island, NY, USA; #78510) with DTT, proteinase inhibitors and phosphatase inhibitors.

### 4.4. Par3 Quantification

After immunostaining for Par3 and Ph-Vimentin, z-stacks of phospho-vimentin+ mitosis were taken with a Zeiss LSM5 confocal. Using Adobe Photoshop software, the level of Par3 and DAPI signals in both daughter cells were quantified in at least 4 consecutive plans. The normalized ratios of fluorescent intensity for both Par3 and DAPI were calculated as described by Bultje et al. [47]. Mitosis that presented a DAPI normalized ratio twice above the average of the DAPI normalized ratio were excluded; Mitosis with a Par3 normalized ratio twice above the average of the DAPI normalized ratio were considered as asymmetric. The percentage of asymmetric division was calculated as the percentage of mitosis presenting an asymmetric distribution of Par3.

### 4.5. Real-Time PCR (qPCR)

Using PerfectPure RNA Fibrous Tissue Kit (Fisher Scientific), RNA was extracted from the telencephalon of E13.5 WT and p73KO embryos, which had undergone treatment for 4 days with either DMSO vehicle or ICG-001 (660 mg/kg). Reverse transcription was performed using a Quanta Biosciences qScript cDNA Synthesis kit. qPCR was run using Quanta PerfeCTa SYBR Green super Mix for iQ on BioRad Cycler. Primer sequences: PPP4c: 5′-CTTGGTAGAAGAGAGCAACGTG-3′, 5′-CGCCACCTACTCTGAACAGC-3′; SMEK1: 5′-TATGACTTGGCCCTTAGCTTTCA-3′, 5′-ACCTGGTGAGGACATATCATCA-3′. Data were normalized to the reference gene, mouse Gapdh. Relative expression levels were calculated using the 2^-ddCt^ method.

### 4.6. Data Analysis

Numerical data were expressed as the means ± SEM, unless otherwise noted. Student’s *t*-test or One-way ANOVA with a post-hoc Newman–Keuls multiple comparison test was performed as appropriate. *p*-values < 0.05 were considered significant.

## 5. Conclusions

Normal long-term repopulating somatic stem cells (SSCs) preferentially divide asymmetrically, with one daughter cell remaining in the niche and the other going on to be a transient amplifying cell required to generate new tissue in homeostatic maintenance and repair processes, whereas cancer stem cells (CSCs) favor symmetric divisions. Even when treated with the CBP/β-catenin antagonist ICG-001, normal SSCs that divide asymmetrically will always retain one of the dividing daughter cells in the stem cell niche. However, when treated with ICG-001, CSCs will be removed from their niche via forced stochastic symmetric differentiative divisions. We now demonstrate that loss of p73 in early corticogenesis biases β-catenin Kat3 coactivator usage and enhances β-catenin/CBP transcription at the expense of β-catenin/p300 transcription. Biased β-catenin coactivator usage has dramatic consequences on the mode of division of neural stem cells (NSCs), but not neurogenic progenitors. The observed increase in symmetric divisions due to enhanced β-catenin/CBP interaction and transcription leads to an increase in NSC symmetric differentiative divisions. Moreover, we demonstrate for the first time that the complex phenotype caused by KO of p73 can be rescued in utero by treatment with the small-molecule-specific CBP/β-catenin antagonist, ICG-001. Taken together, our results demonstrate the causal relationship between the choice of β-catenin Kat3 coactivator and the mode of stem cell division, with major implications for how to therapeutically target and eliminate CSCs, as well as enhance the repair potential of normal SSCs, without damaging or depleting the normal endogenous stem cell populations.

## Figures and Tables

**Table 1:** Percentage of asymmetric mitoses in p73 knock-out (KO) versus wild-type (WT) mice treated with CBP/catenin antagonist versus control (DMSO).

Condition	# Experiments (# Embryos)	# Mitoses	% Asm	Statistic
DMSO-WT	3(7)	63	41.3	(a)
DMSO-KO	3(6)	66	16.7	*
ICG-001-WT	3(10)	74	40.5	(a)
ICG-001-KO	3(10)	72	45.8	ns

Asm, Asymmetric divisions; (a) Unpaired *t*-test; * *p* < 0.05; ns, not significant; #, number.

**Table 2:** Percentage of asymmetric mitoses in wild-type mice (WT) or Tis21-GFP mice treated with CBP/catenin antagonist or p300/catenin antagonist versus control (DMSO). # Mitoses corresponds to the number of mitoses analyzed.

Condition	# Experiments (# embryos)	# Mitoses	%Asm	Statistic
DMSO-WT	6(17)	153	46.4	(a)
ICG-001 (3x)-WT	3(11)	87	47.1	ns
YH249-WT	4(11)	87	6.9	**
YH249 Rescue-WT	3(7)	52	36.5	ns
IQ-1-WT	4(11)	90	22.2	*
DMSO-Tis21-GFP	4(7)	32	65.6	(b)
YH249-Tis21-GFP	3(8)	29	56.7	ns

Asm, Asymmetric divisions; (a) One-way ANOVA; (b) Unpaired *t*-test, ** *p* < 0.01; * *p* < 0.05; ns, not significant; #, number.

## Supplementary Materials

The following are available online at https://www.mdpi.com/2072-6694/11/7/962/s1. Figure S1: Overall cellularity of the cerebral cortex is not affected by p73 knock-out or by specific blockage of the p300/β-catenin interaction, Figure S2: p73 knock-out mouse embryos show an increase in the CBP/β-catenin interaction, Figure S3: p73 regulates PP4, a key factor in mitotic spindle orientation, Figure S4: Prematurely enhanced neurogenic commitment observed with p300/catenin antagonist IQ-1 treatment is reversed by co-administration of CBP/catenin antagonist ICG-001, Table S1: Comparison of PPP4c and SMEK1 mRNA expression in p73 Knock-out (KO) versus Wild-type Mice Treated with CBP/catenin Antagonist versus Control (DMSO).
