# Supplementary material for: The Mode of Stem Cell Division Is Dependent on the Differential Interaction of β-Catenin with the Kat3 Coactivators CBP or p300
Source: Cancers (Basel). 2019 Jul 9;11(7):962. doi: 10.3390/cancers11070962 (PMC6678591; doi:10.3390/cancers11070962)
Supplement: Supplementary file 1 [file cancers-11-00962-s001.pdf]

Supplementary Materials: The Mode of Stem Cell Division Is Dependent on the Differential Interaction of β-Catenin with the Kat3 Coactivators CBP or p300

Agnes I. Lukaszewicz, Cu Nguyen, Elizabeth Melendez, David P. Lin, Jia-Ling Teo, Keane K. Y. Lai, Wieland B. Huttner, Song-Hai Shi and Michael Kahn

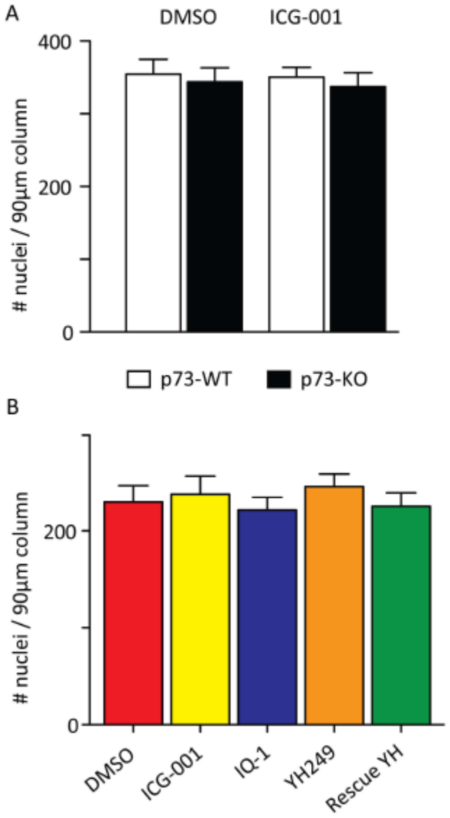

Figure S1. Overall cellularity of the cerebral cortex is not affected by p73 knock-out or by specific blockage of the p300/β-catenin interaction. Cellularity was calculated as the number of DAPI+ nuclei under 90 µm wide cortical column. E13.5 p73 wild type (WT) and knock-out (KO) embryos treated for 4 days with DMSO present with cellularity similar to E13.5 p73 WT and KO embryos treated with CBP/catenin antagonist ICG-001 for 4 days (**A**). E12.5 embryos treated for 3 days with DMSO, ICG-001, p300/catenin antagonist IQ-1, p300/catenin antagonist YH249 or both YH249 and ICG-001 (Rescue YH) show similar cellularity (**B**). $n \geq 6$.

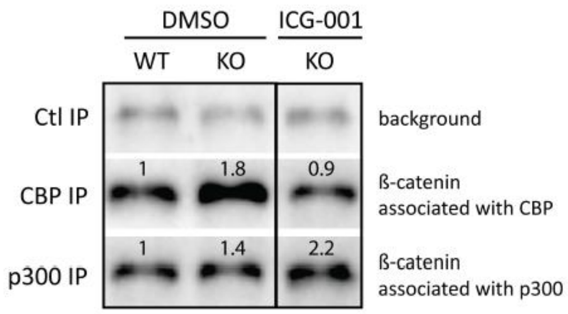

Figure S2. p73 knock-out mouse embryos show an increase in the CBP/β-catenin interaction. Numerical values above bands indicate densitometric quantitation normalized to background (Ctl IP) for each treatment condition and then normalized to WT DMSO for CBP IP or p300 IP.

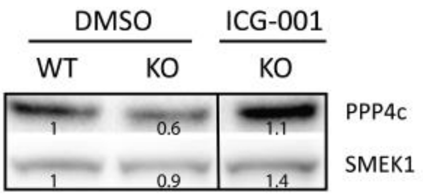

Figure S3. p73 regulates PP4, a key factor in mitotic spindle orientation. Numerical values below bands indicate densitometric quantitation normalized to WT DMSO for PPP4c or SMEK1.

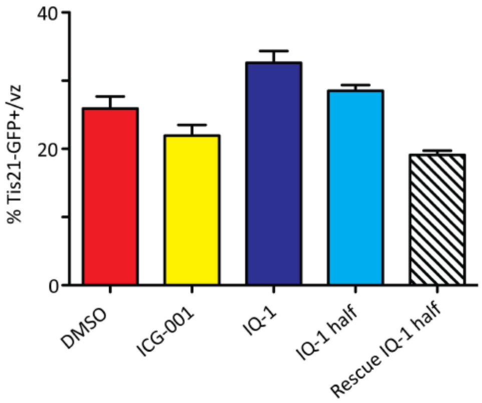

Figure S4. Prematurely enhanced neurogenic commitment observed with p300/catenin antagonist IQ-1 treatment is reversed by co-administration of CBP/catenin antagonist ICG-001. Quantification of Tis21-GFP+ cells as a measure of neurogenic commitment. Embryos were treated for 3 days with DMSO, CBP/catenin antagonist ICG-001, full or half dose of p300/catenin antagonist IQ-1 or IQ-1 and ICG-001 (Rescue IQ-1 half). Sections were stained for GFP and NeuN. The percentage of Tis21-GFP+ cells in the ventricular zone was calculated as the number of GFP+ within the NeuN-negative zone. One-way ANOVA followed by Newman-Keuls multiple comparison test showing significant differences between ICG-001 and both IQ-1 conditions, as well as between the rescue condition and both IQ-1 conditions. vz, ventricular zone. $n \geq 3$.

Table S1. Comparison of PPP4c and SMEK1 mRNA expression in p73. Knock-out (KO) versus Wild-type Mice Treated with CBP/catenin Antagonist versus Control (DMSO).

Condition	PPP4c (ΔΔCt)	SMEK1 (ΔΔCt)
WT<br>ICG-001/DMSO<br>($n = 2/n = 4$)	−0.11	0.29
DMSO<br>KO/WT<br>($n = 3/n = 4$)	−0.26	0.008
ICG-001<br>KO/WT<br>($n = 4/n = 3$)	0.22	0.14

© 2019 by the authors. Licensee MDPI, Basel, Switzerland. This article is an open access article distributed under the terms and conditions of the Creative Commons Attribution (CC BY) license (http://creativecommons.org/licenses/by/4.0/).
